# Familial Hypercholesterolemia in the Arabian Gulf Region: Clinical results of the Gulf FH Registry

**DOI:** 10.1371/journal.pone.0251560

**Published:** 2021-06-04

**Authors:** Khalid F. Alhabib, Khalid Al-Rasadi, Turky H. Almigbal, Mohammed A. Batais, Ibrahim Al-Zakwani, Faisal A. Al-Allaf, Khalid Al-Waili, Fahad Zadjali, Mohammad Alghamdi, Fahad Alnouri, Zuhier Awan, Abdulhalim J. Kinsara, Ahmed AlQudaimi, Wael Almahmeed, Hani Sabbour, Mahmoud Traina, Bassam Atallah, Mohammed Al-Jarallah, Ahmad AlSarraf, Nasreen AlSayed, Haitham Amin, Hani Altaradi

**Affiliations:** 1 Department of Cardiac Sciences, College of Medicine, King Saud University, Riyadh, Saudi Arabia; 2 Medical Research Centre, Sultan Qaboos University, Muscat, Oman; 3 Department of Biochemistry, College of Medicine & Health Sciences, Sultan Qaboos University, Muscat, Oman; 4 Department of Family and Community Medicine, College of Medicine, King Saud University, Riyadh, Saudi Arabia; 5 Alfarabi College of Medicine, Alfarabi Colleges, Riyadh, Saudi Arabia; 6 Department of Pharmacology & Clinical Pharmacy, College of Medicine and Health Sciences, Sultan Qaboos University, Muscat, Oman; 7 Gulf Health Research, Muscat, Oman; 8 Department of Medical Genetics, Faculty of Medicine, Umm Al-Qura University, Makkah, Saudi Arabia; 9 Department of Clinical Biochemistry, Sultan Qaboos University Hospital, Muscat, Oman; 10 National Guard Hospital, Riyadh, Saudi Arabia; 11 Cardiovascular Prevention Unit, Prince Sultan Cardiac Centre, Riyadh, Saudi Arabia; 12 Clinical Biochemistry Department, Faculty of Medicine, King Abdulaziz University, Jeddah, Saudi Arabia; 13 Ministry of National Guard Health Affair, COM-WR, King Abdullah International Medical Research Center, King Saud Bin Abdulaziz University for Health Sciences, Riyadh, Saudi Arabia; 14 Saud Al Babtain Cardiac Center, Dammam, Saudi Arabia; 15 Heart and Vascular Institute, Cleveland Clinic Abu Dhabi, Abu Dhabi, UAE; 16 Department of Pharmacy, Cleveland Clinic Abu Dhabi, Al Maryah Island, Abu Dhabi, United Arab Emirates; 17 Cleveland Clinic Lerner College of Medicine of Case Western Reserve University, Cleveland, OH, United States of America; 18 Department of Medicine, Sabah Al-Ahmed Cardiac Center, Kuwait; 19 Gulf Medical & Diabetes Center, Manama, Bahrain; 20 Bahrain Defence Force Hospital, Riffa, Bahrain; Nagoya University, JAPAN

## Abstract

**Background and aims:**

Familial hypercholesterolemia (FH) is a common autosomal dominant disorder that can result in premature atherosclerotic cardiovascular disease (ASCVD). Limited data are available worldwide about the prevalence and management of FH. Here, we aimed to estimate the prevalence and management of patients with FH in five Arabian Gulf countries (Saudi Arabia, Oman, United Arab Emirates, Kuwait, and Bahrain).

**Methods:**

The multicentre, multinational Gulf FH registry included adults (≥18 years old) recruited from outpatient clinics in 14 tertiary-care centres across five Arabian Gulf countries over the last five years. The Gulf FH registry had four phases: 1- screening, 2- classification based on the Dutch Lipid Clinic Network, 3- genetic testing, and 4- follow-up.

**Results:**

Among 34,366 screened patient records, 3713 patients had suspected FH (mean age: 49±15 years; 52% women) and 306 patients had definite or probable FH. Thus, the estimated FH prevalence was 0.9% (1:112). Treatments included high-intensity statin therapy (34%), ezetimibe (10%), and proprotein convertase subtilisin/kexin type 9 inhibitors (0.4%). Targets for low-density lipoprotein cholesterol (LDL-C) and non-high-density lipoprotein cholesterol were achieved by 12% and 30%, respectively, of patients at high ASCVD risk, and by 3% and 6%, respectively, of patients at very high ASCVD risk (*p* <0.001; for both comparisons).

**Conclusions:**

This snap-shot study was the first to show the high estimated prevalence of FH in the Arabian Gulf region (about 3-fold the estimated prevalence worldwide), and is a “call-to-action” for further confirmation in future population studies. The small proportions of patients that achieved target LDL-C values implied that health care policies need to implement nation-wide screening, raise FH awareness, and improve management strategies for FH.

## Introduction

Familial hypercholesterolemia (FH) is a common, life-threatening genetic disorder. FH is characterized by impaired clearance of low-density lipoprotein cholesterol (LDL-C) from blood, due to mutations in one or more genes, which results in lifelong elevated LDL-C [1]. The autosomal dominant inherited form is typically due to a mutation in the low-density lipoprotein receptor (LDLR), apolipoprotein B (ApoB), or proprotein convertase subtilisin/kexin type 9 (PCSK9). Mutations in the gene codes for the low-density lipoprotein receptor adaptor protein (LDLRAP1) results in the autosomal recessive form of FH [2]. FH can be heterozygous (frequently less severe, due to a single mutated allele) or homozygous (commonly severe, due to mutations in both alleles). Less commonly, FH can also be double heterozygous (two different mutated alleles at two separate genetic loci) or compound heterozygous (two different mutated alleles at one particular gene locus) [3, 4]. In most studied populations, heterozygous FH (HeFH) affects one in 200–500 persons. Homozygous FH (HoFH) is rare; it affects one in 160,000–300,000 persons [5, 6].

Individuals with FH are at high risk of atherosclerosis and its consequences. In those cases, FH manifests as premature coronary artery disease (CAD), and patients are at increased risk of peripheral artery disease and stroke, which increases morbidity and mortality [7]. Although the high CAD risk among patients with FH is well-established, the onset of cardiovascular events is variable; it depends on age, gender, and other risk factors, like hypertension, smoking, and high lipoprotein(a) levels. Previous studies reported that 50% of male patients and 30% of female patients with HeFH developed CAD by the age of 60 years [8]. However, recent studies reported an even higher CAD prevalence at the same age [9].

Various studies have reported that FH is underdiagnosed and undertreated. Among the estimated 34 million individuals with FH, only 1% are identified in most countries [10–12]. Therefore, a reduction in cardiovascular disease (CVD) risk requires early detection and management of FH, particularly in the presence of premature atherosclerotic cardiovascular disease (ASCVD). These measures are particularly important among high-risk populations, like those in the Arabian Gulf countries, where FH prevalence might be high, due to high consanguinity rates [13–15].

Although FH leads to fatal cardiovascular outcomes, data are currently limited worldwide; only 9% of countries in the world have reported the FH prevalence in the general population [16]. Therefore, this study aimed to estimate the prevalence and management of patients with FH in five Arabian Gulf countries.

## Methods

### Study design and population

The study design and detailed methodology of the Gulf FH registry construction were previously published [17]. Briefly, the Gulf FH registry study was a multicentre, multinational study with cross-sectional and prospective components. Participants were recruited from outpatient (primary care, cardiology, endocrinology, and lipid) clinics in 14 tertiary-care centres across five countries in the Arabian Gulf region: 7 centres in Saudi Arabia, 1 centre in Oman, 2 centres in the United Arab Emirates, 2 centres in Kuwait, and 2 centres in Bahrain. The registry had four phases: 1- screening, 2- classification based on the Dutch Lipid Clinic Network (DLCN), 3- genetic testing, and 4- follow-up (S1 Appendix). The present study focused on the first two phases. Results from the follow-up and genetic testing will be reported separately.

Inclusion criteria for enrolment in the Gulf FH registry were: age ≥18 years; Arabian Gulf national; either LDL-C ≥4.9 mmol/L (≥190 mg/dL) or total cholesterol (TC) ≥7.5 mmol/L (≥290 mg/dL); either not taking lipid-lowering treatments or a corrected LDL-C ≥4.9 mmol/L (≥190 mg/dL); and a previous genetic diagnosis of FH. Lipid results were collected retrospectively from hospital medical records over the last five years. Therefore, fasting status was unknown, though most laboratories typically collected lipid samples under fasting conditions. Exclusion criteria were: triglycerides >5 mmol/L (442 mg/dL), history of untreated hypothyroidism, proteinuria ≥1 g/L, obstructive liver disease, chronic kidney disease, human immune deficiency virus infection, and the use of immunosuppressants, steroids, or psychiatric medications.

### Study objectives and definitions

The main objectives of the present Gulf FH registry-based study were to estimate the prevalence and management of patients with FH in five Arabian Gulf countries. The DLCN criteria were used to categorize the patients into one of four FH groups: definite FH (DFH), probable FH (PrFH), possible FH (PoFH), or unlikely FH [18] (S2 Appendix). To calculate the FH prevalence, we merged data for patients with DFH and PrFH and excluded 132 patients with confirmed genetic mutations as these patients were recruited from highly specialized lipid clinics in only two countries in the Gulf region (Saudi Arabia and Oman). Patients had a family history of premature CVD when a first-degree relative had premature CVD (male <55 years old or female <65 years old) [19]. Statin therapy was classified as: high-intensity, moderate-intensity, or low-intensity statin [20] (S3 Appendix). Based on the 2016 guidelines established by the European Society of Cardiology and the European Atherosclerosis Society (ESC/EAS), lipid targets for LDL-C were <2.6 mmol/L (<100 mg/dL) and <1.8 mmol/L (<70 mg/dL), for those at high and very-high ASCVD risk, respectively. The 2016 lipid targets for non-HDL-C were <3.4 mmol/L (<130 mg/dL) and <2.6 mmol/L (<100 mg/dL) for those at high and very-high ASCVD risk, respectively [20]. In the updated, 2019 version of the ESC/EAS dyslipidaemia guidelines, lipid targets for LDL-C were <1.8 mmol/L (<70 mg/dL) and <1.4 mmol/L (<55 mg/dL) for those at high and very-high ASCVD risk, respectively. The 2019 lipid targets for non-HDL-C were <2.6 mmol/L (<100 mg/dL) and <2.2 mmol/L (<85 mg/dL) for those at high and very-high ASCVD risk, respectively [21].

Based on the ESC/EAS 2019 dyslipidaemia guidelines, patients with FH were considered at very high risk of ASCVD, when ASCVD was documented, or when another major risk factor was present; patients with FH were considered at high risk of ASCVD, when no other major CVD risk factors were present. ASCVD was diagnosed (either based on clinical or unequivocal imaging evidence) when patients had previous evidence of acute coronary syndrome (i.e., myocardial infarction or unstable angina), stable angina, coronary revascularization (percutaneous coronary intervention [PCI], coronary artery bypass graft, or other arterial revascularization procedures), stroke or transient ischaemic attack, or peripheral artery disease. Unequivocal imaging evidence of ASCVD included findings known to be predictive of clinical events, such as a significant plaque (i.e., multivessel coronary disease, with two major epicardial arteries >50% stenotic) detected with coronary angiography, computerized tomography (CT), or carotid ultrasound [21].

### Study organization and ethical approval

The study was organized by the co-principal investigators, national leaders, co-investigators, and study manager of the five included countries in the Arabian Gulf region. The study was approved by the following ethical commettees and institutional review boards:

1. King Saud University Medical City IRB.
2. King Abdullah International Medical Research Center IRB.
3. Cardiac Research Department, Prince Sultan Cardiac Center.
4. Saud Albabtain Cardiac Center IRB.
5. King Abdulaziz University Ethics Committee.
6. Sheikh Khalifa Medical City IRB.
7. Alain Hospital Research and Ethics Governance Committee.
8. Gulf Diabetes Specialist Center Ethics Committee.
9. Kuwait MOH Research Ethics Committee.
10. Research Ethics Committee Royal Medical Services, Bahrain Defence Force.
11. Sultan Qaboos University Hospital Ethics Committee.

### Statistical analysis

The sample size was calculated, based on a conservative estimate that the HeFH prevalence would be 1:500 [17] (S4 Appendix). We estimated a 10% snap-shot sample of the expected HeFH proportion of the populations in each of the five Arabian countries. The final calculated sample size was 5179. The study steering committee considered that at least 70% of the calculated sample size (n = 3625) would be sufficient, in view of the expected missing or unclear data.

Descriptive statistics are expressed as the frequency and percentage for categorical variables. Differences between the FH groups were analysed with Pearson’s χ2 test (or Fisher’s exact test for <5 expected cells). Continuous variables are expressed as the mean and standard deviation (±SD). Statin daily doses are expressed as the median and interquartile range (IQR). Differences between groups were analysed with the Kruskal-Wallis test. An *a priori* two-tailed level of significance was set at 0.05. Statistical analyses were performed with STATA version 13.1 (STATA Corporation, College Station, TX, USA).

## Results

### Prevalence of FH

**Fig 1:**
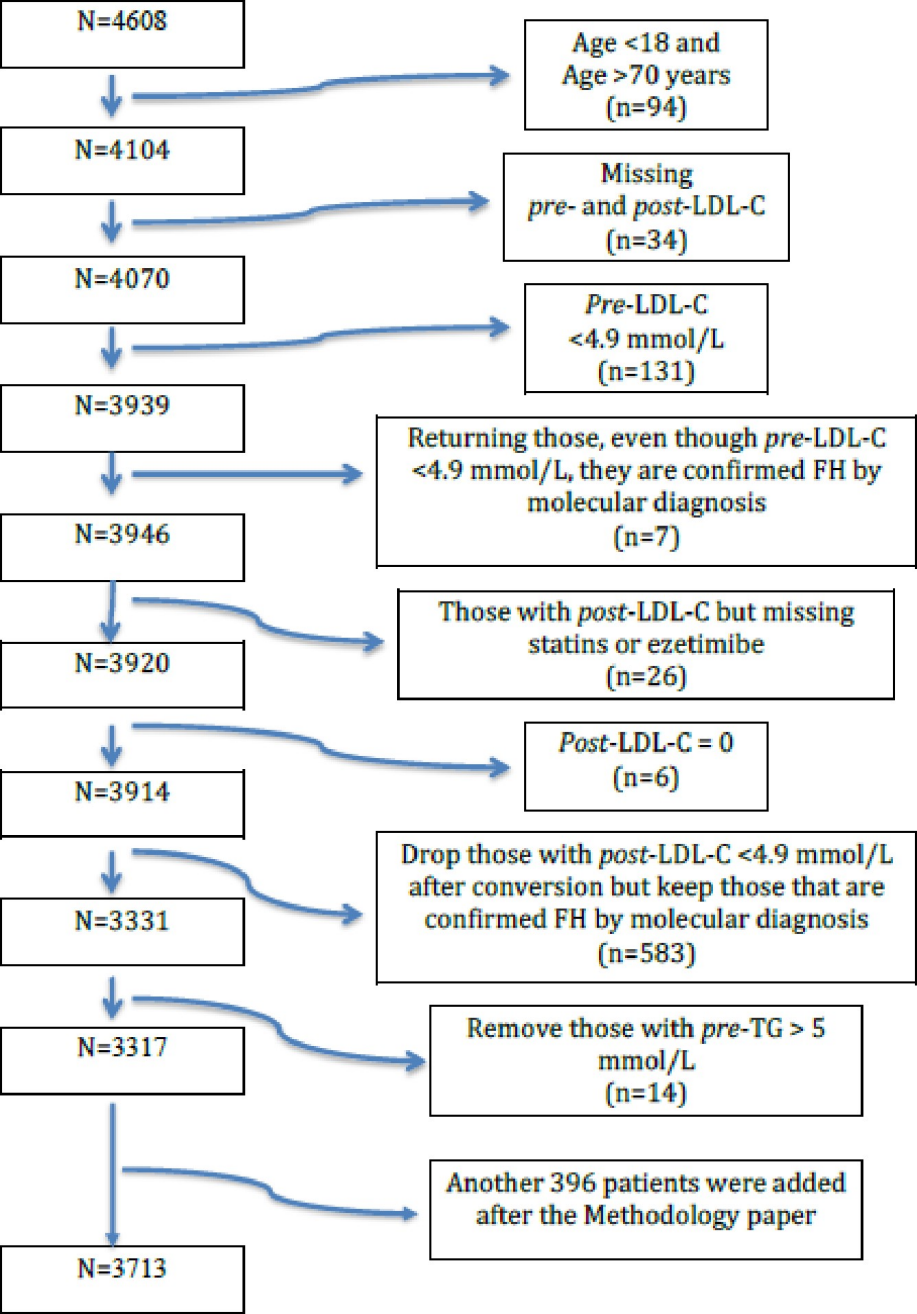
Schematic diagram of patient flow of Gulf FH study. *This figure is adapted from the Methodology paper, with only one exception, being the addition of 396 patients thereafter.

Among 34,366 participants screened and was filtered further based on the inclusion/exclusion criteria and the missing essential data for the DLCN criteria (Fig 1) Schematic diagram of patient flow of Gulf FH study, the final data consisted of 3,713 patients which was higher than the cohort in the initial published Gulf FH design paper as more patients have been included [17]. After stratifying the cohort, based on the DLCN criteria, there were 195 (5.3%) patients with DFH, 243 (6.5%) patients with PrFH, 2801 (75.4%) patients with PoFH, and 474 (12.8%) patients with unlikely FH. Thus, among all screened participants, the FH prevalence (including both PrFH and DFH groups) was 0.9% (306/34,234, i.e., 1:112).

### Patient demographics and clinical characteristics

The overall mean age±SD of the patients was 49±15 years, and 1948 (52%) were women (Table 1). A positive history of FH was documented in the hospital medical records of 1181 (32%) patients, based on either clinical or genetic results. The prevalence of diabetes mellitus was higher among patients with PoFH (28%) or unlikely FH (28%), compared to those with PrFH/DFH (23%; *p* <0.001). However, compared to those with PoFH or unlikely FH, more patients with PrFH/DFH had histories of premature CAD (33% vs. 8.4% and 6.3%, respectively; *p* <0.001), angina (19% vs. 5.5% and 3.4%, respectively; *p* <0.001), myocardial infarction (14% vs. 4.1% and 3.4%, respectively; *p* <0.001), premature cerebrovascular diseases (3.9% vs. 1.5% and 1.5%, respectively; *p* = 0.001), and premature peripheral artery disease (1.4% vs. 0.4% and 0.2%, respectively; p = 0.032). Smoking was reported by 278 (7.5%) patients. Tendon xanthomas were present in 55 (13%) patients with PrFH/DFH and absent in all individuals with PoFH or unlikely FH. Histories of cardiac procedures and investigations (i.e., PCI, coronary artery bypass graft, CT angiogram, CT coronary calcium score, and echocardiography) were found more frequently among patients with PrFH/DFH than among patients with PoFH or unlikely FH (Table 1).

**Table 1:** Demographic and clinical characteristics of the Gulf FH cohort, stratified by the Dutch Lipid Clinic Network (DLCN) criteria.

Characteristic	All (N = 3713)	DLCN			*p*-value
		Unlikely FH	PoFH	PrFH/DFH	
		n = 474; 12.8%	n = 2801; 75.4%	n = 438; 11.8%	
*Demographic*					
Age, mean±SD, years	49±15	50±13	48±13	51±23	<0.001
Female gender	1948 (52%)	257 (54%)	1501 (54%)	190 (43%)	<0.001
Smoking	278 (7.5%)	27 (5.7%)	182 (6.5%)	69 (16%)	<0.001
*Medical history*					
History of FH*	1181 (32%)	64 (14%)	832 (30%)	285 (65%)	<0.001
Tendon xanthomas	55 (1.5%)	0	0	55 (13%)	<0.001
Arcus corneal	122 (3.3%)	3 (0.6%)	48 (1.7%)	71 (16%)	<0.001
Diabetes mellitus	1024 (28%)	132 (28%)	790 (28%)	102 (23%)	<0.001
Hypertension	779 (21%)	99 (21%)	585 (21%)	95 (22%)	0.927
Hx of premature CAD	411 (11%)	30 (6.3%)	236 (8.4%)	145 (33%)	<0.001
Hx of angina	254 (6.8%)	16 (3.4%)	153 (5.5%)	85 (19%)	<0.001
Hx of MI	191 (5.1%)	16 (3.4%)	114 (4.1%)	61 (14%)	<0.001
Hx of premature cerebrovascular diseases	65 (1.8%)	7 (1.5%)	41 (1.5%)	17 (3.9%)	0.001
Hx of premature PAD	18 (0.5%)	1 (0.2%)	11 (0.4%)	3 (1.4%)	0.032
*Procedures & Investigations*					
PCI	237 (6.4%)	16 (3.4%)	136 (4.9%)	85 (19%)	<0.001
CABG	106 (2.9%)	4 (0.8%)	56 (2.0%)	46 (11%)	<0.001
Hx of CT angiogram	81 (2.2%)	11 (2.3%)	38 (1.4%)	32 (7.3%)	<0.001
Hx of CT coronary calcium score	78 (2.1%)	8 (1.7%)	37 (1.3%)	33 (7.5%)	<0.001
Hx of echocardiography	722 (19%)	64 (14%)	497 (18%)	161 (37%)	<0.001
Hx of carotid doppler	85 (2.3%)	6 (1.3%)	51 (1.8%)	28 (6.4%)	<0.001

Values are the number of subjects (%), unless specified otherwise. Hx, history; MI, myocardial infarction; PAD, peripheral artery disease; CABG, coronary artery bypass graft.

Percentages might not sum to 100% due to rounding.

### Medical therapy

**Fig 2:**
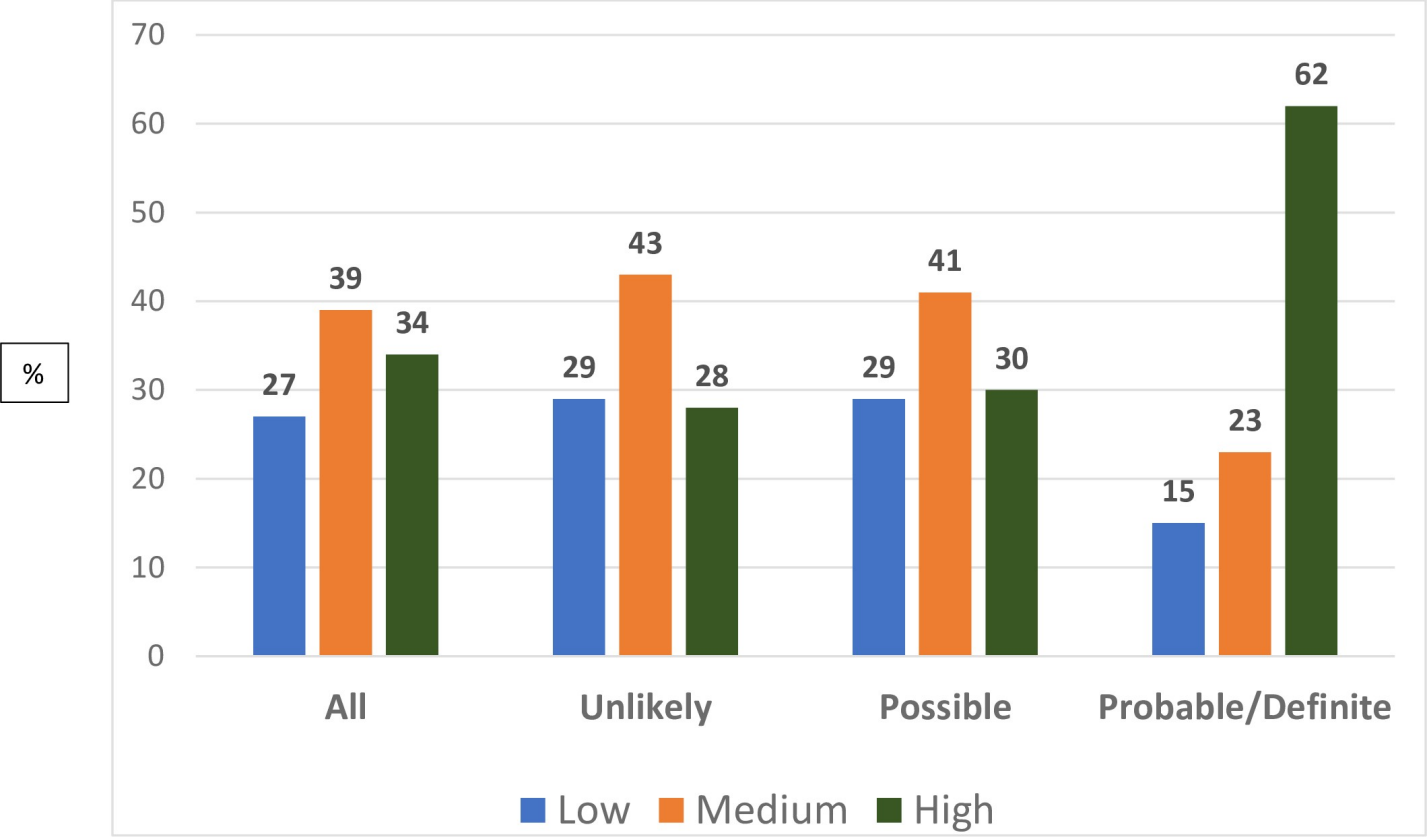
Intensity of statin therapy among the Gulf familial hypercholesterolemia cohort stratified by the Dutch Lipid Clinic Network (DLCN). **High-intensity statin therapy was defined as those on atorvastatin 40–80 mg and rosuvastatin 20–40 mg while medium-intensity statin therapy was defined as those on atorvastatin 10–20 mg, rosuvastatin 5–10 mg, simvastatin 20–40 mg and pravastatin 40–80 mg. Low intensity statin therapy was defined as those on simvastatin 10 mg or pravastatin 10–20 mg.

A total of 3192 (86%) participants used lipid-lowering medications. Atorvastatin, rosuvastatin, simvastatin, and pravastatin were prescribed for 1426 (45%), 777 (24%), 237 (7.4%), and 2 (0.1%) patients, respectively. Patients with PrFH/DFH were more likely (62%) to be treated with high-intensity statin treatments than those with PoFH (30%) or unlikely FH (28%; *p* <0.001; (Fig 2) Intensity of statin therapy among the Gulf familial hypercholesterolemia cohort stratified by the Dutch Lipid Clinic Network (DLCN). Among patients with PrFH/DFH that were managed with lipid-lowering treatments, almost one-third (33%) were treated with ezetimibe, 6 (1.4%) were treated with PCSK9 inhibitors, and 12 (2.8%) underwent LDL-apheresis (Table 2).

**Table 2:** Medication status the Gulf FH cohort, stratified by the Dutch Lipid Clinic Network (DLCN) criteria.

Medications, daily dose (mg)	All (N = 3192)	DLCN			*p*-value
		Unlikely FH	Po FH	PrFH/DFH	
		(n = 401)	(n = 2359)	(n = 432)	
Simvastatin	237 (7.4%)	35 (8.7%)	182 (7.7%)	20 (4.6%)	0.036
Median (IQR)	20 (20, 20)	20 (20, 40)	20 (20, 20)	20 (20, 40)	0.089
Atorvastatin	1426 (45%)	210 (52%)	1030 (44%)	186 (43%)	0.004
Median (IQR)	20 (20, 40)	20 (20, 40)	20 (20, 40)	40 (20, 40)	<0.001
Rosuvastatin	777 (24%)	57 (14%)	551 (23%)	169 (39%)	<0.001
Median (IQR)	20 (10, 40)	20 (10, 30)	20 (10, 20)	40 (20, 40)	<0.001
Pravastatin	2 (0.1%)	0	2 (0.1%)	0	1.000
Median (IQR)	30 (20, 40)	0	30 (20, 40)	0	n/a
Ezetimibe	326 (10%)	30 (7.5%)	155 (6.6%)	141 (33%)	<0.001
Fenofibrate	21 (0.7%)	3 (0.8%)	12 (0.5%)	6 (1.4%)	0.096
Gemfibrozil	9 (0.3%)	0	8 (0.3%)	1 (0.2%)	0.852
Omega 3	23 (0.7%)	1 (0.3%)	19 (0.8%)	3 (0.7%)	0.607
Aspirin	736 (23%)	77 (19%)	498 (21%)	161 (37%)	<0.001
PCSK9 inhibitor	14 (0.4%)	2 (0.5%)	6 (0.3%)	6 (1.4%)	<0.001
		Evoloc	3*Aliroc	4*Evoloc	
			3*Evoloc	2*Aliroc	
Diabetic treatment	809 (25%)	101 (25%)	630 (27%)	78 (18%)	0.001
LDL-apheresis	15 (0.5%)	1 (0.3%)	2 (0.1%)	12 (2.8%)	<0.001

Values are the number of subjects (%), unless specified otherwise; n/a, not applicable

* = treated with; Evoloc, Evolocumab; Aliroc, Alirocumab. Among 3713 subjects in the full cohort, 521 (14%) were missing information on medications. Percentages might not sum to 100%, due to rounding.

### Patient lipid profiles

Mean TC levels at baseline and post-treatment, respectively, were: PrFH/DFH: 9.54 and 7.12 mmol/L, PoFH: 7.67 and 5.84 mmol/L, and unlikely FH: 6.71 and 5.33 mmol/L (Table 3). Mean LDL-C levels at baseline and post-treatment, respectively, were: PrFH/DFH: 7.61 and 5.96 mmol/L, PoFH: 5.68 and 4.09 mmol/L, and unlikely FH: 4.70 and 3.54 mmol/L. All of these differences between baseline and post-treatment levels were statistically significant (*p* <0.001).

**Table 3:** Baseline and post-treatment lipid profiles for the Gulf FH cohort, stratified by Dutch Lipid Clinic Network criteria (DLCN).

Characteristic	All (N = 3713)	DLCN			*p-value*
		Unlikely FH	PoFH	PrFH/DFH	
		(n = 474)	(n = 2801)	(n = 438)	
Total cholesterol^a^					
Baseline	7.70±1.24	6.71±0.80	7.67±0.89	9.54±2.49	<0.001
Post-treatment	6.01±1.86	5.33±2.33	5.84±1.29	7.12±2.78	<0.001
LDL-cholesterol^b^					
Baseline	5.71±1.09	4.70±0.51	5.68±0.68	7.61±2.36	<0.001
Post-treatment	4.36±2.04	3.54±1.28	4.09±1.27	5.96±3.55	<0.001
Non-HDL-cholesterol^c^					
Baseline	6.45±1.24	5.37±0.77	6.42±0.85	8.34±2.61	<0.001
Post-treatment	4.80±1.90	4.10±2.33	4.62±1.30	5.94±2.87	<0.001

Values are the mean concentration (mmol/L)±standard deviation.

^a^2996 and 1978 patients had populated total cholesterol levels at baseline and follow-up, respectively

^b^3004 and 1974 patients had non-missing LDL-cholesterol levels at baseline and follow-up, respectively.

^c^ 2923 and 1941 patients had non HDL-C levels at baseline and follow-up, respectively.

### LDL-C and non-HDL-C target achievements

**Fig 3:**
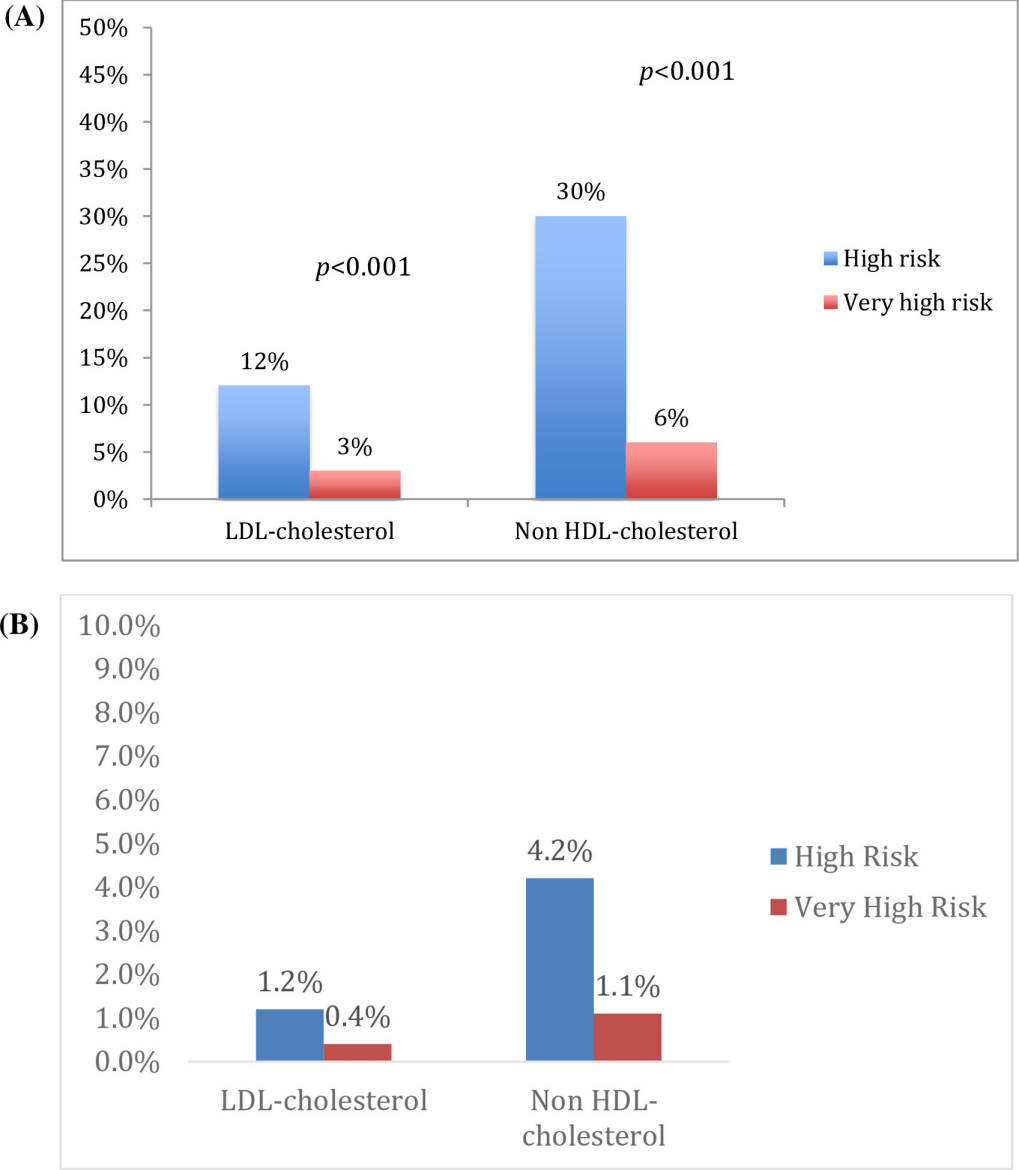
LDL and non HDL cholesterol goal attainments in the Gulf FH cohort, stratified by ASCVD risk. (A) Percentages of subjects that achieved the ESC/EAS-2016 lipid targets. LDL-cholesterol targets were <2.6 mmol/L and <1.8 mmol/L, for the high and very high ASCVD risk groups, respectively; non HDL-cholesterol targets were 3.4 mmol/L and 2.6 mmol/L, for the high and very high ASCVD risk groups, respectively. (B) Percentages of subjects that achieved the ESC/EAS-2019 lipid targets. LDL-cholesterol targets were <1.8 mmol/L and <1.4 mmol/L, for the high and very high ASCVD risk groups, respectively; no HDL-cholesterol targets were <2.6 mmol/L and <2.2 mmol/L, for the high and very high ASCVD risk groups, respectively.

Most patients did not achieve the lipid targets. LDL-C and non-HDL-C targets were achieved by 12% and 30%, respectively, in the high ASCVD risk group, and by 3% and 6%, respectively, in the very-high ASCVD risk group (between-group differences: *p* <0.001 for both targets; (Fig 3) [21]. LDL and non HDL cholesterol goal attainments in the Gulf FH cohort, stratified by ASCVD risk. (A) Percentages of subjects that achieved the ESC/EAS-2016 lipid targets. LDL-cholesterol targets were <2.6 mmol/L and <1.8 mmol/L, for the high and very high ASCVD risk groups, respectively; non HDL-cholesterol targets were 3.4 mmol/L and 2.6 mmol/L, for the high and very high ASCVD risk groups, respectively. (B) Percentages of subjects that achieved the ESC/EAS-2019 lipid targets. LDL-cholesterol targets were <1.8 mmol/L and <1.4 mmol/L, for the high and very high ASCVD risk groups, respectively; non HDL-cholesterol targets were <2.6 mmol/L and <2.2 mmol/L, for the high and very high ASCVD risk groups, respectively.

## Discussion

This Gulf FH registry-based study was the first multinational study to estimate the prevalence and management of FH in adult patients in the Arabian Gulf region. We revealed a high prevalence of FH (1:112) in a snap-shot sample taken from various specialized and non-specialized out-patient clinics in the region. Most participants with PrFH/DFH had a history of FH and a history of ASCVD. In addition, patients with PrFH/DFH could not adequately achieve LDL-C or non-HDL-C targets.

Our study results, and the results of other recent international registries, supported the notion that the true FH prevalence is most likely higher than the previously reported prevalence of 1:250–500 [22, 23]. The FH prevalence (1:112) we found in the Gulf FH registry was about 3-fold of the estimated worldwide FH prevalence recently published in two meta-analyses that included 11 million (1:313) and over 7.3 million individuals (1:311) [16, 24]. Although there is a high consanguinity rate in the Gulf region (as high as 50%) [13–15], the high prevalence of FH is likely due to founder effect [25]. Furthermore, recruiting patients from tertiary-care centres might have led to including a population with FH that was in relatively worse health than the general population; hence, a relatively high FH prevalence could be expected. Despite these caveats, FH has been underdiagnosed and undertreated worldwide, as shown by previous findings from other registries [26]. This oversight could be due to a lack of national registries, difficulty in performing genetic analyses, due to either unavailability and/or prohibitive costs, a lack of specialized lipid clinics, and a paucity of educational programs to raise physician awareness of FH. A recent Saudi study that investigated physician FH awareness, practices, and knowledge showed that 93% of participants had poor knowledge of FH [27]. However, several courses and symposia have been conducted recently in the Arabian Gulf region in collaboration with the International Atherosclerosis Society. That endeavour aimed to raise awareness of the diagnosis and management of patients with FH [28, 29].

The prevalences of diabetes mellitus, hypertension, and smoking were 23%, 22%, and 16%, respectively, among patients with PrFH/DFH. These results were similar to the high prevalences of these modifiable CVD risk factors in the general population of the Arabian Gulf region [30–32]. Although these findings were generally similar to those in other international FH registries, few studies have reported that the prevalence of these modifiable CVD risk factors was lower among patients with FH than among the general population. For example, a large-scale observational study, conducted as part of the national Dutch screening program, reported a 50% lower risk of diabetes mellitus among individuals with FH compared to the general population [33]. A plausible explanation could be that patients with FH might adopt relatively healthier lifestyles to minimize CVD risk [34].

The reported CAD prevalence among those with PrFH/DFH in our study (33%) was consistent with the prevalence rates of CAD in patients with FH in other international registries [35, 36]. For example, CAD prevalences among patients with FH were 35.9%, in the US CASCADE-FH Registry, and 38.8% in a UK study [37, 38]. Moreover, patients with FH are at high risk of ASCVD and require intensive management with lipid-lowering treatments [39].

Large LDL-C reductions in patients with FH have been associated with reduced CAD progression and reductions in CVD events and mortality rates [40–43]. The lipid target achievements in our study were poor. Only 12% and 3% of patients achieved the 2016 LDL-C targets (<2.6 mmol/L and <1.8 mmol/L) in the high ASCVD risk and very-high ASCVD risk groups, respectively. Although the majority (86.8%) of patients with PrFH/DFH in our study received statins, only about two-thirds (62%) received high-intensity doses; 33% received ezetimibe, 2.8% underwent LDL apheresis, and 1.4% received PCSK9 inhibitors. These results were similar to those reported in other registries, with only slight differences. In the American CASCADE-FH registry, only 25% of adult subjects with FH achieved the LDL-C target (LDL-C <2.6 mmol/L); 75% received statins, 42% received high-intensity statins, and 34% received statins and ezetimibe [36]. In the Netherlands, another FH registry study showed that 21% of the cohort achieved the LDL-C target (LDL-C <2.6 mmol/L), 96% received statins, 34% received high-intensity statins, and 53% received statins and ezetimibe [44].

The low rates of achieving the target LDL-C could have been due to inadequate prescribing, and/or the use of high-intensity statins, ezetimibe, or PCSK9 inhibitors (individually or in combination, due to cost and/or side effects), and/or low compliance with a healthy lifestyle [45, 46]. Adding ezetimibe to statin therapy can decrease LDL-C levels by 60–70% in individuals with FH [47]. LDL-C can be reduced by 50–70% with lipoprotein apheresis, but that therapy is expensive, and it is only available in very few centres in the Arabian Gulf region [48]. Recently, PCSK9 inhibitors (alirocumab and evolocumab) were shown to lower LDL-C by around 60% (when added to lipid lowering therapies, mainly statins), and they improved CVD outcomes in patients with FH [49–51]. Nevertheless, the rate of PCSK9 inhibitor use remains low in our region, mainly due to the high cost. However, studies have shown that these medications were cost-effective, at a threshold of $100,000 per quality-adjusted life-year (QALY), when the annual price was ≤$4536 (45). Currently, the annual price in the Arabian Gulf region remains above the cost-effective price, at $5962 [52].

This study had various strengths, including the first-of-a-kind registry conducted in five Arabian Gulf counties. Moreover, we used standard DLCN criteria for diagnosing FH and well-defined exclusion criteria to avoid confounding with other potential causes of dyslipidaemia.

This study also had some limitations. First, its retrospective design had inherent limitations, such as missing data and uncertainties about compliance to medical therapies and healthy lifestyles. Data were not available for the time period between the measurements of baseline and post-treatment serum cholesterol levels. However, it was likely that most patients had been taking lipid-lowering therapies for several months and even years. Moreover, our study included only adults 18 years or older; hence, any inferences to FH prevalence or management would not be applicable to children. In addition, we might have overestimated the prevalence of FH, because participants were recruited from outpatient clinics in tertiary-care centres; thus, a potential referral bias could have resulted in a patient population with relatively poor health and more severe LDL-C levels than expected in the general population. However, the included centres were flagship hospitals from different health care sectors and geographic regions that had wide catchment areas, with referrals from the surrounding districts and even from outside their respective cities. Having said that, the results of our snap-shot study should be interpreted with caution, and to be viewed as a “call-to-action” for the Ministries of Health to confirm our findings in a nation-wide registry program in the Arabian Gulf population.

In conclusion, we showed that the Arabian Gulf region had a high estimated prevalence of adults with FH (3-fold the estimated prevalence worldwide), and a low proportion of patients achieved the target LDL-C. Our results have major implications for healthcare policy makers, with regard to establishing national programs and policies for nation-wide FH screening (index screening and cascade screening for first-degree relatives of patients with FH), raising FH awareness, and improving FH management. In future, the Gulf FH registry will report on detailed genetics and 1-year clinical follow-ups for patients with FH.

## Supporting information

S1 Appendix The four Gulf FH registry phases. (DOCX)

S2 Appendix Dutch Lipid Clinic Network (DLCN) criteria. (DOCX)

S3 Appendix Intensities of statin treatment. (DOCX)

S4 Appendix Sample size calculations. (DOCX)

S5 Appendix Study coordinators for the Gulf FH registry. (DOCX)

S6 Appendix International research collaborators for the Gulf FH registry. (DOCX)
