# Supplementary material for: Familial Hypercholesterolemia in the Arabian Gulf Region: Clinical results of the Gulf FH Registry
Source: PLoS One. 2021 Jun 4;16(6):e0251560. doi: 10.1371/journal.pone.0251560 (PMC8177652; doi:10.1371/journal.pone.0251560)
Supplement: S1 Appendix — (DOCX) [file pone.0251560.s001.docx]

**S1 Appendix. The four Gulf FH Registry phases.**

| **No** | **Phase** | **Description** |
| --- | --- | --- |
| **1** | **Screening** | Patients with potential FH that met the eligibility criteria were recruited, based on their lipid profile and medical data collected from the hospital information system in the last 5 years. Patients taking lipid-lowering treatments were included when the corrected LDL-C was ≥4.9 mmol/L (≥190 mg/dl), based on a well-known correction formula. Electronic case report forms were completed by the site investigators after obtaining the final data. |
| **2** | **Classification; based on DLCN criteria** | Patients without a previous genetic diagnosis were classified according to DLCN criteria as follows: unlikely FH, possible FH (PoFH), probable FH (PrFH), and definitive FH (DFH). Of those, PrFH, and DFH were taken as FH in the present study. |
| **3** | **Genetic testing** | Around 500 patients with FH were selected for genetic testing to sequence the four common FH-related genes (*LDLR*, *APOB*, *PCSK9*, and *LDKLRAP1*). |
| **4** | **Follow-up** | Patients that underwent genetic testing and patients with previous genetic test results were followed for 1 year to evaluate their medical management and CVD outcomes. |

LDL-C: low-density lipoprotein cholesterol; DLCN: Dutch Lipid Clinic Network; LDLR: LDL receptor; APOB: apolipoprotein B; PCSK9: proprotein convertase subtilisin/kexin type 9; LDKLRAP: LDLR adaptor protein; CVD: cardiovascular disease.
