# Supplementary figures and images for: Familial Hypercholesterolemia in the Arabian Gulf Region: Clinical results of the Gulf FH Registry
Source: PLoS One. 2021 Jun 4;16(6):e0251560. doi: 10.1371/journal.pone.0251560 (PMC8177652; doi:10.1371/journal.pone.0251560)

**S2 Appendix**
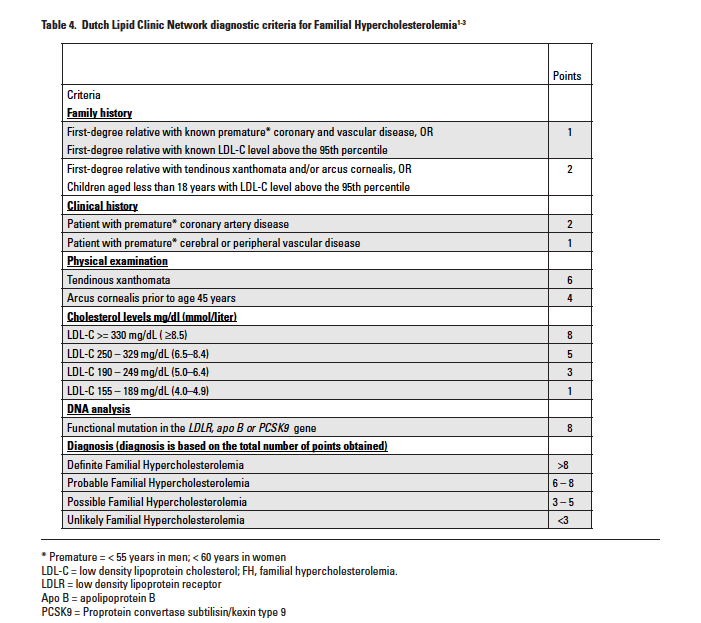
**. Dutch Lipid Clinic Network (DLCN) criteria.**

Supplement: S2 Appendix — (DOCX) [file pone.0251560.s002.docx]

**S3 Appendix. Intensities of statin treatment.**


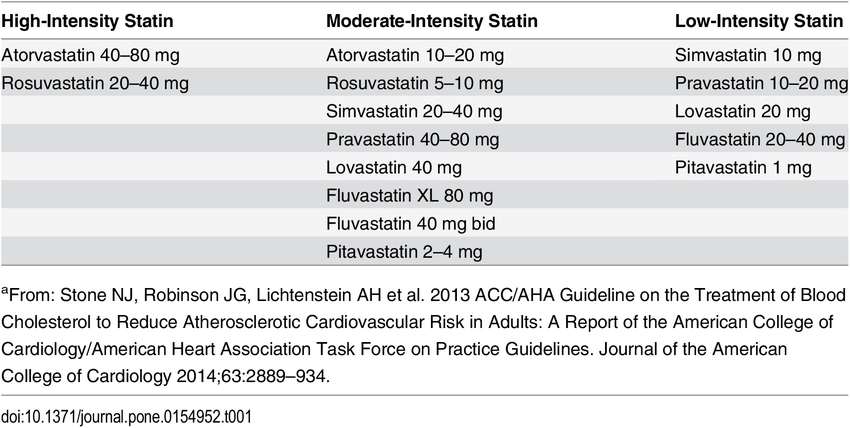

Supplement: S3 Appendix — (DOCX) [file pone.0251560.s003.docx]
