# Supplementary material for: Familial Hypercholesterolemia in the Arabian Gulf Region: Clinical results of the Gulf FH Registry
Source: PLoS One. 2021 Jun 4;16(6):e0251560. doi: 10.1371/journal.pone.0251560 (PMC8177652; doi:10.1371/journal.pone.0251560)
Supplement: S4 Appendix — (DOCX) [file pone.0251560.s004.docx]

**S4 Appendix. Sample size calculations.**

| **Country** | **National population** | **Expected HeFH (1:500)** | **Sample size as 10% of the regional sample** |
| --- | --- | --- | --- |
| **Bahrain** | **630,990** | **1262** | **126** |
| **Kuwait** | **1,281,712** | **2563** | **256** |
| **Oman** | **2,323,954** | **4648** | **465** |
| **Saudi Arabia** | **20,708,462** | **41,417** | **4,142** |
| **United Arab Emirates** | **950,368** | **1901** | **190** |
| **Total** | **25,895,486** | **51,791** | **5179** |

**HeFH, heterozygous familial hypercholesterolaemia.**
