# Supplementary material for: Familial Hypercholesterolemia in the Arabian Gulf Region: Clinical results of the Gulf FH Registry
Source: PLoS One. 2021 Jun 4;16(6):e0251560. doi: 10.1371/journal.pone.0251560 (PMC8177652; doi:10.1371/journal.pone.0251560)
Supplement: S5 Appendix — (DOCX) [file pone.0251560.s005.docx]

**S5 Appendix. Study coordinators for the Gulf FH registry.**

| **Country** | **Names** |
| --- | --- |
| **Saudi Arabia** | Kazi Nur Asfina, Mohammad Athar, Attayeb Alameen, Iyad Farah, Fahmi Alkaf, Alia Khudary, Nawal Alrowaily, Omran Rashidi, Emad Nogali, Emad Sindi, Mansour Altayyar, Dina Nuwaylati, Roa Bamanie, Ashraf Hammouda (Deceased) , Hind Al Kammar, Attieah Saleh, Ariane Poral, Marhemat Palmansali, Mohammad Al Bagshy, Majedah Al-Maraj, Omer A Elamin |
| **Oman** | Suad Al Mukhaini |
| **UAE** | Abdullah Shehab, Dana Abdul Hai, Naema Luqman, Arshad Abdulrasheed |
| **Kuwait** | Mirjana Radovic |
| **Bahrain** | Gozde Yasayan |
