# Supplementary material for: Familial Hypercholesterolemia in the Arabian Gulf Region: Clinical results of the Gulf FH Registry
Source: PLoS One. 2021 Jun 4;16(6):e0251560. doi: 10.1371/journal.pone.0251560 (PMC8177652; doi:10.1371/journal.pone.0251560)
Supplement: S6 Appendix — (DOCX) [file pone.0251560.s006.docx]

**S6 Appendix. International research collaborators for the Gulf FH registry.**

| **Name** | **Affiliation** |
| --- | --- |
| **Raul D. Santos** | Associate Professor and Director of the Lipid Clinic Heart Institute (InCor), University of Sao Paulo Medical School Hospital, Sao Paulo, Brazil  Researcher at the Hospital Israelita Albert Einstein  President of the International Atherosclerosis Society |
| **Kausik K. Ray** | Department of Public Health and Primary Care,  Imperial Centre for Cardiovascular Disease  Prevention, Imperial College London, London, UK |
